# Alien flora of Zimbabwe: Data derived from herbarium specimens

**DOI:** 10.1016/j.dib.2022.108186

**Published:** 2022-04-21

**Authors:** Maroyi Alfred

**Affiliations:** Department of Botany, University of Fort Hare, Alice, South Africa

**Keywords:** Alien plants, Conservation, Gardens, Exotic species, Horticulture, Zimbabwe

## Abstract

This data is based on herbarium specimens housed by the National Herbarium (SRGH) in Harare, Zimbabwe. Comprehensive data is provided for 1487 alien plant taxa growing in Zimbabwe. This checklist has 1027 taxa that were confined to cultivation, while 63 and 397 taxa were recorded as casuals and naturalized, respectively. The alien flora is rich in Fabaceae *sensu lato* (13.7%), Asteraceae and Poaceae (7.3% each) plant families. The dataset presented in this article provide important baseline information on alien plant species in Zimbabwe required to monitor status of such species within the country, particularly in protected areas. Therefore, this dataset has potential to serve as a foundation for future research on plant invasions in Zimbabwe.

## Specifications Table

**Table:** 

Subject	Biological sciences, biodiversity
Specific subject area	Botany, conservation, alien plants, invasive species
Type of data	Tables, figures, database, map
How data were acquired	Data set was generated from herbarium specimens, field work and alien plant surveys conducted throughout the country aimed at establishing the spatial distribution of alien species in Zimbabwe, their distributional range, habitats and abundance [1], [2], [3], [4], [5], [6], [7], [8], [9]
Data format	Raw data in Excel file, analyzed
Description of data collection	This dataset has a list of alien species occurring in Zimbabwe was compiled based on herbarium records, field work and alien plant surveys conducted throughout the country [1], [2], [3], [4], [5], [6], [7], [8], [9]. All alien plant species ever recorded in Zimbabwe, whether the introduction was accidental or intentional, as escapes from cultivation or naturalized at least once in the wild were included in this study. Each species was assessed to ascertain its current residential status in the country. The habitats from which the species were recorded were compiled from herbarium specimen labels and this information is provided for each species. Processed data divided alien species recorded in Zimbabwe into three main categories following methodologies to generate the level of naturalization [10]: (i): species that were determined to occur only in cultivation, (ii): species that were recorded as escapes or that are casual aliens, and (iii): species that were recorded as naturalized.
Data source location	National Herbarium, Harare, Zimbabwe.
Data accessibility	The data is available with this article.

## Value of the Data

• Data set presented provide a comprehensive baseline data on alien flora of Zimbabwe. All alien plant species ever recorded in Zimbabwe were included in the database as cultivated, casual or naturalized. Several species currently used as food plants, ornamentals, shade, fodder, sources of timber and fimber in Zimbabwe were introduced in the country in the 19th and 20th centuries. Some of these species now occupy large areas of land in Zimbabwe with significant number of these species categorized as invasive species. Species invasions are one of the main conservation threats worldwide and have caused many species extinctions.
• The data is of value to plant taxonomists and conservation biologists who are interested in species invasions, impacts of alien species on ecosystem modifications, monitoring and management of alien species. The dataset is a crucial starting point as important alien species have been identified together with their associated habitats. Such data will enable plant taxonomists and conservation biologists to monitor new introductions and manage existing alien species in the country. This data will enable researchers to analyze the floristic status, biological attributes and geographical distribution of alien species growing in Zimbabwe as a stratergy for understanding the ecological relationships between the invader and the invaded habitats. This dataset will also enable future researchers to study ecological and socio-economic impacts of alien species on the native plant communities in Zimbabwe.
• The data also identifies alien species that are likely to be problem plants or invasive in the near future. The dataset has alien plant species that have potential to suppress or replace indigenous species. The dataset therefore, emphasize the alien species and the problems associated with alien invasions. A better understanding of alien plant establishment, spread, socio-economic, ecological and distributional changes are important for making informed decisions about alien plants.
• The sampling technique of using data derived from herbarium specimens, field work and alien plant surveys enable future researchers to determine the association between alien species and the environment, particularly, soil, climate, landscape and anthropogenic factors.
• This dataset provides baseline data required by Zimbabwe to establish and implement a cost effective, objective and sound alien plants monitoring system in the country.

## 1 Data Description

**Fig. 1:**
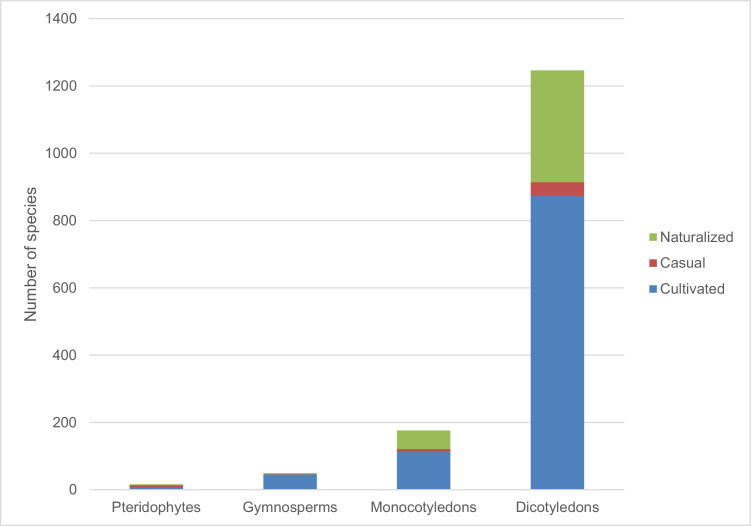
Number of plant species recorded as introduced in Zimbabwe.

**Fig. 2:**
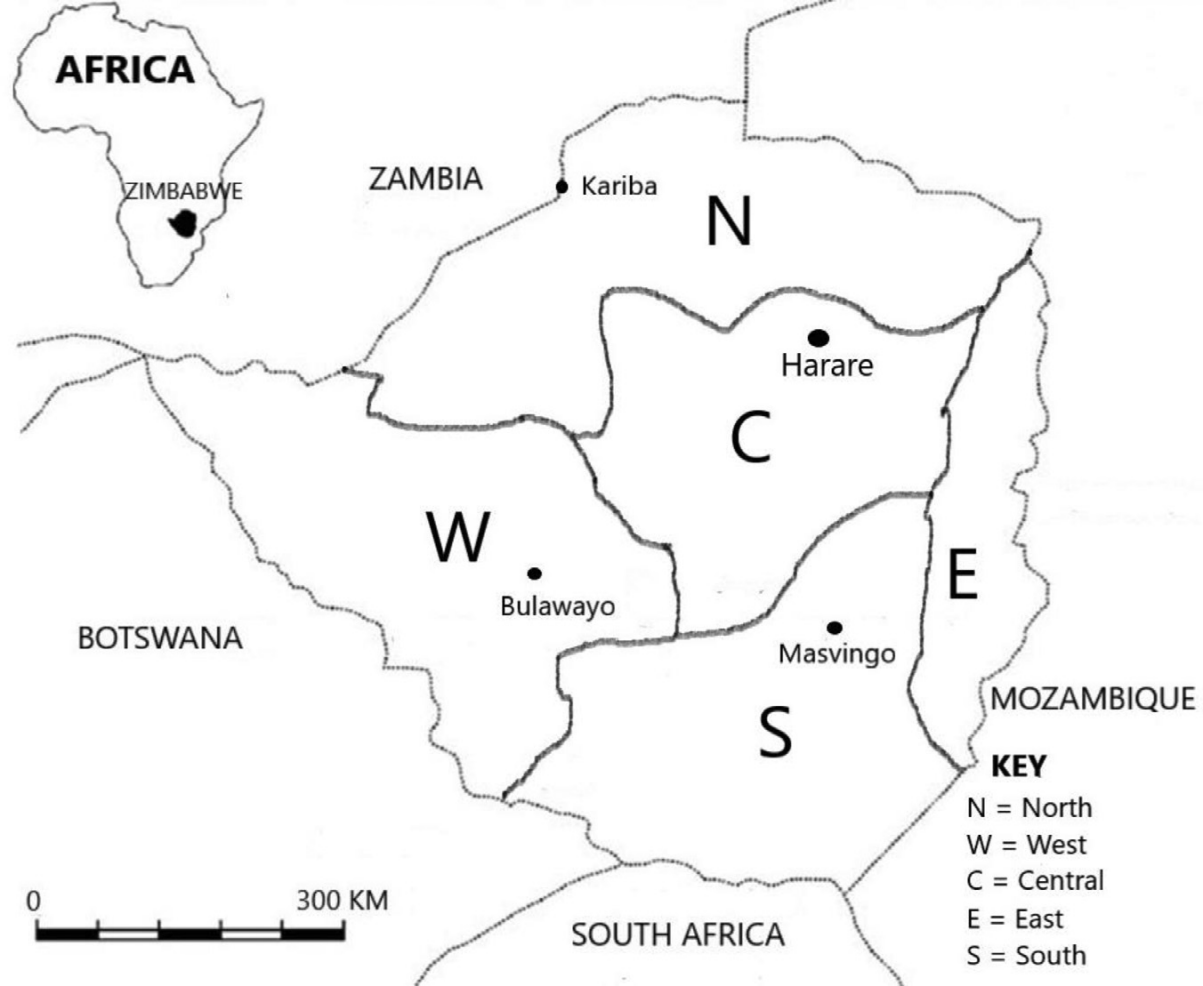
Number of plant species recorded as introduced in Zimbabwe.

**Fig. 3:**
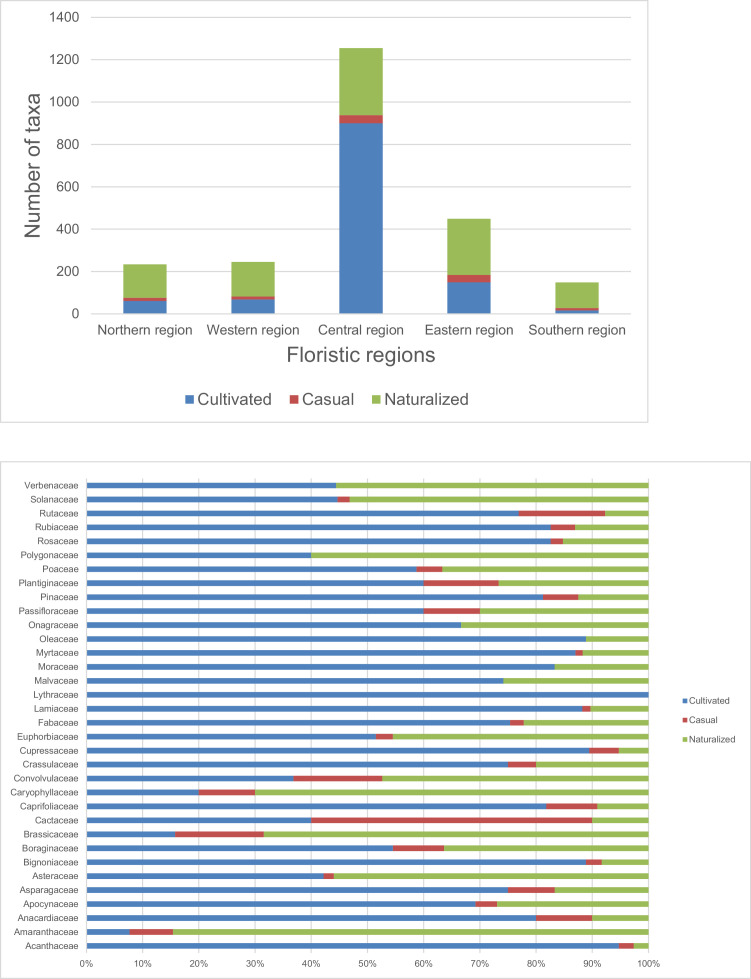
Families with 10 or more introduced species.

**Fig. 4:**
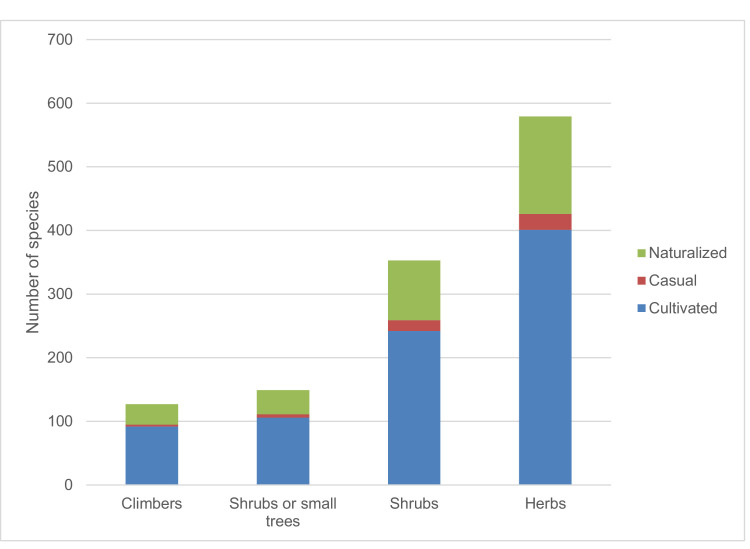
Life forms of recorded introduced plant species in Zimbabwe.

The data include 1487 taxa (Appendix 1), 16 pteridophytes, 50 gymnosperms, 176 monocotyledons and 1245 dicotyledons (Fig. 1). Of these, 1027 species are confined to cultivation, while 63 species were recorded as casuals and 397 were recorded as naturalized. Among the documented plant families, Fabaceae *sensu lato* accounted for the highest number of introduced species (203), Asteraceae and Poaceae (109 species each), Myrtaceae (77 species), Lamiaceae (68 species), Solanaceae (47 species), Rosaceae (46 species), Acanthaceae (38 species), Bignoniaceae (36 species), Euphorbiaceae (33 species), Malvaceae (31 species), Amaranthaceae and Apocynaceae (26 species each), Rubiaceae (23 species), Crassulaceae (20 species), Brassicaceae, Convolvulaceae and Cupressaceae (19 species each), Moraceae, Oleaceae and Verbenaceae (18 species each), Pinaceae (16 species), Onagraceae, Plantiginaceae and Polygonaceae (15 species each), Rutaceae (13 species), Asparagaceae (12 species), Boraginaceae and Caprifoliaceae (11 species each), Anacardiaceae, Cactaceae, Caryophyllaceae, Lythraceae and Passifloraceae (10 species each) (Fig. 2). The life form distribution of documented species is shown in Fig. 3, Fig. 4. Considerable effort has been made to provide a comprehensive catalog of the alien flora of Zimbabwe [1], [2], [3]. There is still need to indicate the naturalization of each alien taxon. The current study categorized the species as cultivated, casual and naturalized. This categorization is based on Maroyi [4] who argued that casual aliens reproduce occasionally outside cultivation, do not form self-sustaining populations and rely on repeated introductions for their persistence while naturalized species are aliens that reproduce consistently without direct human intervention.

## 2 Experimental Design, Materials and Methods

A list of alien plants to Zimbabwe was compiled from Herbarium specimens housed by the National Herbarium (SRGH) in Harare, Zimbabwe. All alien plant species ever recorded in Zimbabwe, whether the introduction was accidental or intentional, as escapes from cultivation or naturalized at least once in the wild were included in this study. Some taxa were determined to occur only in cultivation, while others were recorded as escapes, casual aliens or naturalized after Pyšek et al. [10]. Pyšek et al. [10] defined a casual alien as a species that reproduce occasionally outside cultivation and do not form self-sustaining populations and rely on repeated introductions for their persistence. Similarly, the same authors defined a naturalized species as an alien species that reproduce consistently without direct human intervention. Each species was assessed to ascertain its current residential status in the country through herbarium studies, field work and alien plant surveys conducted throughout the country aimed at establishing the spatial distribution of alien species in Zimbabwe, their distributional range, habitats and abundance [1], [2], [3], [4], [5], [6], [7], [8], [9]. The processed data divided alien species recorded in Zimbabwe into three main categories following methodologies to generate the level of naturalization [10]:

(i) species that were determined to occur only in cultivation,
(ii) species that were recorded as escapes or that are casual aliens, and
(iii) species that were recorded as naturalized.

This data is a revision of a preliminary checklist of introduced plant species in Zimbabwe compiled in 1996 [1] as several species have been introduced into the country since this date and there was also need to provide up to date and valid plant names. The taxon names conform to those of the Plants of the World Online [11]. The plant families and genera are listed alphabetically under Pteridophyta, Gymnospermae and Angiospermae, the latter being subdivided into monocotyledons and dicotyledons (Appendix 1). Each species’ distribution in Zimbabwe is indicated by letters showing the floristic divisions used in Flora Zambesiaca region, that is, Northern region (N), Western region (W), Central region (C), Eastern region (E) and Southern region (S) [12].

## Ethics Statement

No ethical approval for this study was required as the studied herbarium specimens were freely available in the National Herbarium.

## CRediT authorship contribution statement

**Maroyi Alfred:** Conceptualization, Methodology, Data curation, Supervision, Writing – review & editing.

## Declaration of Competing Interest

The authors declare that they have no known competing financial interests or personal relationships that could have appeared to influence the work reported in this paper.

## Data Availability

A preliminary checklist of naturalized and introduced plants in Zimbabwe.
